# Metabolic syndrome in pregnancy and risk for adverse pregnancy outcomes: A prospective cohort of nulliparous women

**DOI:** 10.1371/journal.pmed.1002710

**Published:** 2018-12-04

**Authors:** Jessica A. Grieger, Tina Bianco-Miotto, Luke E. Grzeskowiak, Shalem Y. Leemaqz, Lucilla Poston, Lesley M. McCowan, Louise C. Kenny, Jenny E. Myers, James J. Walker, Gus A. Dekker, Claire T. Roberts

**Affiliations:** 1 Robinson Research Institute, University of Adelaide, Adelaide, Australia; 2 Adelaide Medical School, University of Adelaide, Adelaide, Australia; 3 Waite Research Institute, School of Agriculture, Food and Wine, University of Adelaide, Adelaide, Australia; 4 Department of Women and Children’s Health, King’s College London, St. Thomas’ Hospital, London, United Kingdom; 5 Department of Obstetrics and Gynaecology, University of Auckland, Auckland, New Zealand; 6 Faculty of Health & Life Sciences, University of Liverpool, Liverpool, United Kingdom; 7 Maternal and Fetal Health Research Centre, University of Manchester, Manchester, United Kingdom; 8 Obstetrics and Gynaecology Section, Leeds Institute of Biomedical and Clinical Sciences, University of Leeds, Leeds, United Kingdom; 9 Women and Children’s Division, Lyell McEwin Hospital, Adelaide, Australia; University of Cambridge, UNITED KINGDOM

## Abstract

**Background:**

Obesity increases the risk for developing gestational diabetes mellitus (GDM) and preeclampsia (PE), which both associate with increased risk for type 2 diabetes mellitus (T2DM) and cardiovascular disease (CVD) in women in later life. In the general population, metabolic syndrome (MetS) associates with T2DM and CVD. The impact of maternal MetS on pregnancy outcomes, in nulliparous pregnant women, has not been investigated.

**Methods and findings:**

Low-risk, nulliparous women were recruited to the multi-centre, international prospective Screening for Pregnancy Endpoints (SCOPE) cohort between 11 November 2004 and 28 February 2011. Women were assessed for a range of demographic, lifestyle, and metabolic health variables at 15 ± 1 weeks’ gestation. MetS was defined according to International Diabetes Federation (IDF) criteria for adults: waist circumference ≥80 cm, along with any 2 of the following: raised trigycerides (≥1.70 mmol/l [≥150 mg/dl]), reduced high-density lipoprotein cholesterol (<1.29 mmol/l [<50 mg/dl]), raised blood pressure (BP) (i.e., systolic BP ≥130 mm Hg or diastolic BP ≥85 mm Hg), or raised plasma glucose (≥5.6 mmol/l). Log-binomial regression analyses were used to examine the risk for each pregnancy outcome (GDM, PE, large for gestational age [LGA], small for gestational age [SGA], and spontaneous preterm birth [sPTB]) with each of the 5 individual components for MetS and as a composite measure (i.e., MetS, as defined by the IDF). The relative risks, adjusted for maternal BMI, age, study centre, ethnicity, socioeconomic index, physical activity, smoking status, depression status, and fetal sex, are reported. A total of 5,530 women were included, and 12.3% (*n =* 684) had MetS. Women with MetS were at an increased risk for PE by a factor of 1.63 (95% CI 1.23 to 2.15) and for GDM by 3.71 (95% CI 2.42 to 5.67). In absolute terms, for PE, women with MetS had an adjusted excess risk of 2.52% (95% CI 1.51% to 4.11%) and, for GDM, had an adjusted excess risk of 8.66% (95% CI 5.38% to 13.94%). Diagnosis of MetS was not associated with increased risk for LGA, SGA, or sPTB. Increasing BMI in combination with MetS increased the estimated probability for GDM and decreased the probability of an uncomplicated pregnancy. Limitations of this study are that there are several different definitions for MetS in the adult population, and as there are none for pregnancy, we cannot be sure that the IDF criteria are the most appropriate definition for pregnancy. Furthermore, MetS was assessed in the first trimester and may not reflect pre-pregnancy metabolic health status.

**Conclusions:**

We did not compare the impact of individual metabolic components with that of MetS as a composite, and therefore cannot conclude that MetS is better at identifying women at risk. However, more than half of the women who had MetS in early pregnancy developed a pregnancy complication compared with just over a third of women who did not have MetS. Furthermore, while increasing BMI increases the probability of GDM, the addition of MetS exacerbates this probability. Further studies are required to determine if individual MetS components act synergistically or independently.

## Abstract 2

Claire Trelford Roberts and colleagues report on the association of metabolic syndrome in pregnant women with increased risk for adverse pregnancy outcomes such as gestational diabetes and preeclampsia.

## Author summary

**Why was this study done?:**

- Obesity increases the risk for developing gestational diabetes mellitus (GDM) and preeclampsia (PE), which both associate with increased risk for type 2 diabetes mellitus (T2DM) and cardiovascular disease (CVD) in women in later life.
- In the general population, metabolic syndrome (MetS) associates with T2DM and CVD.
- The impact of maternal MetS on pregnancy outcomes in nulliparous pregnant women has not been thoroughly investigated.

**What did the researchers do and find?:**

- We assessed the association between MetS, measured at 15 ± 1 weeks’ gestation, and a range of pregnancy complications in low-risk, nulliparous women recruited to the multi-centre, international prospective Screening for Pregnancy Endpoints (SCOPE) study.
- Women with MetS in early pregnancy had an increased risk for GDM and PE, after adjustment for a range of demographic and lifestyle variables.

**What do these findings mean?:**

- Diagnosis of MetS may be useful to broadly identify a group of women at risk for pregnancy complications, which may additionally associate with future CVD risk.
- Future studies are required to substantiate our findings and to determine whether MetS is a useful discriminator of pregnancy complications in lean and obese women. Future studies are also encouraged to define clear metabolic health phenotypes that will produce the optimal probability threshold for each outcome.

## Introduction

Obesity is an established risk factor for pregnancy complications, increasing risk for gestational diabetes mellitus (GDM), preeclampsia (PE), and delivering large for gestational age (LGA) infants, by 2- to 3-fold [1–4], and small for gestational age (SGA) infants, by 24% [5]. Such adverse outcomes place women and their infants at greater risk for cardiovascular and metabolic diseases both perinatally and in later life [6,7]. However, not all obese women experience a pregnancy complication [8], nor do they all develop chronic disease [9].

Metabolic syndrome (MetS) is a cluster of risk factors that encompasses metabolic, vascular, and inflammatory indicators. Although there are several definitions and cut-points used to describe and characterise MetS [10], the metabolic disturbances underpinning MetS are consistent and include atherogenic dyslipidemia, raised blood pressure (BP), insulin resistance, obesity, and pro-thrombotic and pro-inflammatory states. While some expert definitions deem obesity an essential criterion [11,12], other definitions largely focus on insulin resistance [11,13,14]. To date, there are no obligatory components to define MetS [11] but rather a constellation of risk factors that, irrespective of components and cut-offs, have consistently been demonstrated to increase risk for cardiovascular disease (CVD) [15], some cancers [16], type 2 diabetes mellitus (T2DM) [17], and chronic kidney disease [18] in the adult population.

Normal pregnancy is a pro-inflammatory, pro-thrombotic, highly insulin resistant [19], and hyperlipidemic state [20]. However, there are no recognised healthy metabolic variable cut-points in pregnancy. Studies that have assessed metabolic components in pregnancy have generally used accepted definitions for the adult population [21,22] or used a range of population-specific criteria [23–25]. Appropriate definitions that associate metabolic health parameters and pregnancy complications have yet to be defined, and the utility of using previously defined variables from the non-pregnant adult population is unclear.

Given the established links between MetS and chronic diseases in adulthood [15,17], as well as between pregnancy complications such as PE and GDM and later life T2DM and CVD [26,27], pregnancy may offer a window of opportunity to identify women with MetS and elevated risk of adverse pregnancy outcomes, as well as later life chronic disease. Most studies to date, however, have only assessed individual metabolic components in pregnancy: raised triglycerides (TGs) and low-density lipoprotein cholesterol (LDL-C) and reduced high-density lipoprotein cholesterol (HDL-C) are associated with increased risk for GDM, PE, LGA, and spontaneous preterm birth (sPTB) [24,25,28]. In a small study, multiparous Greek women who had MetS, determined in early pregnancy, were at a 3-fold increased risk for preterm birth [29].

Assessment of MetS and a broader range of pregnancy complications in nulliparous women has not been done. We hypothesized that women with MetS are at greater risk for pregnancy complications than women who do not have MetS. The aims of this study were to (i) determine the prevalence of MetS in women participating in the Screening for Pregnancy Endpoints (SCOPE) study and (ii) determine whether MetS is associated with maternal and neonatal outcomes including GDM, LGA, PE, SGA, and sPTB.

## Methods

### Study population

Participants recruited to SCOPE were nulliparous pregnant women recruited from Adelaide (Australia), Auckland (New Zealand), Cork (Ireland), Leeds (UK), London (UK), and Manchester (UK) (*n =* 5,628). SCOPE is a multi-centre prospective cohort study with the primary aim of developing screening tests for prediction of PE, sPTB, and SGA babies. Recruitment was between 11 November 2004 and 28 February 2011, and data collection occurred until 30 September 2011, when the last babies were born. Included women were nulliparous with singleton pregnancies. Women were excluded if they were considered to be at high risk for PE, SGA, or sPTB due to underlying medical conditions (e.g., chronic hypertension requiring antihypertensive medication or diabetes); if they had previous cervical knife cone biopsy; if they had 3 terminations or 3 miscarriages; if their pregnancy was complicated by a known major fetal anomaly or abnormal karyotype; or if they received interventions that may modify pregnancy outcome (e.g., aspirin or cervical suture). Women were also excluded if they were taking supplements of calcium (>1 g/d), eicosopentanoic acid (≥2.7 g/d), vitamin C (>1,000 mg/d), or vitamin E (>400 IU/d) or if they had diabetes (type 1 or type 2). An uncomplicated pregnancy was defined as a normotensive pregnancy, delivered at ≥37 weeks, resulting in a liveborn, non-SGA, and non-LGA baby. Pregnancies with other complications—such as placenta praevia, placental abruption, cholestasis of pregnancy, or other significant pregnancy complication—were not included in the uncomplicated pregnancy group.

Study data were obtained by a research midwife at 15 ± 1 weeks’ gestation, including demographics; smoking; family, medical, and gynaecological history; diet and supplement use; systolic and diastolic BP; height and weight (measured at 15 ± 1 weeks’ gestation, to determine BMI), and waist circumference (WC). The socioeconomic index (SEI) is a measure of the individual’s socioeconomic status and is derived from the specific occupation of the woman, producing a score between 10 and 90, with a lower score reflecting greater disadvantage [30]. The Edinburgh Postnatal Depression Scale was evaluated at 15 weeks’ gestation, and women were categorised as unlikely to experience depression (score <5), at increased risk of depression in the next year (score of 5–9), or likely depressed (score >9). Physical activity at 15 weeks’ gestation was categorised as none (no moderate or vigorous exercise, with ≤1 instance/day of recreational walking), light (no moderate or vigorous exercise, with >1 instance/day of recreational walking), or moderate or vigorous (some moderate or vigorous exercise, with or without recreational walking). Ethnicity was binary, coded as white or other. Smoking status was binary, coded as yes or no for any cigarette smoking at 15 ± 1 weeks’ gestation.

A non-fasting blood sample was taken for measurement of HDL-C and TGs at 15 ± 1 weeks’ gestation. Details of the immunoassay methodology for measuring lipids can be found in previous publications [31,32]. Plasma blood glucose was measured as a random blood sample by glucometer at 15 ± 1 weeks’ gestation. Additional information on data collection has been provided in detail previously [33]. Ethical approval was obtained from local ethics committees (New Zealand AKX/02/00/364; Australia REC 1712/5/2008; London, Leeds, and Manchester 06/MRE01/98; and Cork ECM5 [10] 05/02/08), and all women provided written informed consent. The study is reported as per STROBE guidelines (S1 STROBE Checklist).

### Maternal exposure: MetS

We defined MetS using the International Diabetes Federation (IDF) MetS criteria [12], assessed at 15 ± 1 weeks’ gestation. A WC ≥80 cm is a prerequisite risk factor, along with any 2 of the 4 following variables: raised TGs (≥1.70 mmol/l [≥150 mg/dl]), reduced HDL-C (<1.29 mmol/l [<50 mg/dl]), raised BP (i.e., systolic BP ≥130 mm Hg or diastolic BP ≥85 mm Hg), or raised fasting plasma glucose (≥5.6 mmol/l). As this was a pregnancy cohort, a random plasma glucose was measured. The utility of random plasma glucose has recently been demonstrated in a sample of 25,543 women in the UK, showing that in 69% of these women, random plasma glucose was able to predict GDM (area under the curve [AUC] 0.8) better than maternal BMI (AUC 0.65) and maternal age (AUC 0.60) [34].

### Primary outcome measures: Pregnancy and birth outcomes

PE was defined as systolic BP ≥140 mm Hg or diastolic BP ≥90 mm Hg, or both, on at least 2 occasions at least 4 hours apart after 20 weeks’ gestation but before the onset of labour, or postpartum, with either proteinuria (24-hour urinary protein ≥300 mg or spot urine protein:creatinine ratio ≥30 mg/mmol creatinine or urine dipstick protein ≥ ++) or any multisystem complication of PE [33,35]. GDM was defined using the new World Health Organization classification (fasting glucose of ≥5.1 mmol/l or, following an oral glucose tolerance test, a 2-hour level of ≥8.5 mmol/l) [36]. Universal screening was not employed for GDM in Ireland and the UK; women were only screened if they were identified as being at risk based on factors such as family history and BMI. Therefore, GDM analysis was confined only to women from Australia and New Zealand (*n =* 3,126). sPTB was defined as spontaneous delivery before 37 + 0 weeks’ gestation. Infant measurements (head circumference, length, and birth weight) were recorded by research midwives within 72 hours of birth. Customised birth weight centiles were calculated correcting for gestational age at birth, maternal ethnicity, maternal weight and height in early pregnancy, parity, and infant sex [37]. SGA and LGA were defined as a birth weight <10th or >90th customised centile, respectively.

### Statistical analyses

#### Missing data

For the 5,628 eligible women for whom follow-up data were available, the proportion of missing data for the metabolic variables and the covariates was <2% (S1 Table), such that 97.5% of the data were included in the complete case analysis.

#### Main analyses

Log-binomial regression analyses were used to examine the risk for each pregnancy outcome (GDM, LGA, PE, SGA, and sPTB) with each of the 5 individual components for MetS and as a composite measure. The relative risks, adjusted for maternal BMI, age, study centre (Adelaide, Auckland, Cork, Leeds, London, or Manchester), ethnicity, SEI, physical activity, smoking status, depression status, and fetal sex, are reported. The corresponding 95% CIs for MetS and its components were calculated defined by pd[(RR − 1)/RR] [38], where pd is the proportion of cases with MetS and RR is the adjusted relative risk. Generalised additive models (GAMs) [39], using the GAMLSS R package, were used to examine the risk for each pregnancy outcome by MetS status, with a non-linear penalised spline for BMI and an interaction term between BMI and MetS status, adjusting for potential confounders identified a priori. The statistical software used was R version 3.4.3. The alpha level of significance was set at 5% (i.e., *P* <0.05).

#### Supplementary analyses

Box plots demonstrating median (interquartile range) for each metabolic abnormality, split by MetS status, were drawn, and frequencies (*n*, percent) of women with each pregnancy complication and with each metabolic abnormality were described. To identify the frequency for each combination of MetS components, with and without stratification for BMI, UpSet plots were used to show the overall frequency of each criterion (left bar chart in S3–S5 Figs) and all observed combinations (indicated by dots at the bottom of the figures), sorted from highest to lowest frequency.

#### Analysis formulation

A detailed analysis plan was not written prior to the initiation of the project. However, based on the objectives of this study, we defined all basic analyses to be undertaken in a written document approved by the SCOPE Consortium Scientific Committee (S1 Text), as well as in meetings with all primary authors, including epidemiologists, clinicians, and statisticians, before the start of the analyses. Confounders were identified a priori through the use of directed acyclic graphs. Changes in the analysis plan are described in S1 Text.

## Results

### Descriptive characteristics

A total of 5,530 women were included in the current analysis. S1 Fig shows the participant flow. Overall, 12.3% (*n =* 684) had MetS. Table 1 describes maternal and infant characteristics according to whether women did or did not have MetS. Women with MetS had an approximately 5-kg/m^2^ higher average BMI, had a lower average SEI, and were more likely to smoke, and the mean gestational age at birth was slightly lower.

**Table 1:** Maternal and infant characteristics according to maternal metabolic syndrome, at 15 weeks’ gestation.

Characteristic	Metabolic syndrome		*P* value
	No (*n* = 4,846)	Yes (*n* = 684)	
**Age, years**	28.6 ± 5.4	28.8 ± 5.9	0.5453
**Body mass index, kg/m**^**2**^	24.7 ± 4.5	29.5 ± 5.7	0.0000
**Socioeconomic index**	42.1 ± 16.6	39.4 ± 15.7	0.0001
**Ethnicity**			
White	4,361 (90.0%)	618 (90.4%)	Ref
Other	485 (10.0%)	66 (9.7%)	0.8217
**Study centre (country)**			
Adelaide (Australia)	952 (19.6%)	201 (29.4%)	Ref
Cork (Ireland)	1,572 (32.4%)	192 (28.1%)	0.0000
Auckland (New Zealand)	1,740 (35.9%)	237 (34.6%)	0.0001
Leeds (UK)	132 (2.7%)	12 (1.8%)	0.0391
London (UK)	173 (3.6%)	6 (0.9%)	0.0000
Manchester (UK)	277 (5.7%)	36 (5.3%)	0.0732
**Physical activity**			
None	766 (15.9%)	116 (17.0%)	Ref
Light	317 (6.6%)	40 (5.9%)	0.8004
Moderate or vigorous	3,741 (77.5%)	526 (77.1%)	1.0000
**Smoked at 15 weeks’ gestation**	495 (10.2%)	101 (14.8%)	0.0004
**Depression category**			
Unlikely to be depressed	1,784 (37.0%)	252 (36.9%)	Ref
Increased risk of depression	1,783 (37.0%)	234 (34.3%)	0.9531
Likely to be depressed	1,257 (26.0%)	197 (28.8%)	0.6662
**Fetal sex**			
Male	2,468 (51.1%)	338 (49.6%)	Ref
Female	2,364 (48.9%)	343 (50.4%)	0.5064
**Gestational age at birth, weeks**	39.6 ± 2.5	39.3 ± 2.8	0.0027
**Birth weight, g**	3,402.0 ± 576.5	3,405.1 ± 673.5	0.3839
**Birth weight centile**	47.8 ± 28.9	46.5 ± 30.5	0.2156
**Birth length, cm**	50.3 ± 3.1	50.1 ± 3.4	0.4818
**Head circumference, cm**	34.6 ± 1.9	34.7 ± 2.2	0.0986
**Delivered post-term**			
No (≤41 weeks)	3,804 (78.5%)	546 (79.8%)	Ref
Yes (>41 weeks)	1,042 (21.5%)	138 (20.2%)	0.4575
**Ponderal index**			
Male	2.67 ± 0.41	2.67 ± 0.29	0.7445
Female	2.70 ± 0.34	2.71 ± 0.30	0.5101

Categorical variables are presented as number (percentage), and continuous variables as mean ± standard deviation. All variables (except fetal sex and birth outcomes) were collected at 15 ± 1 weeks’ gestation. *P* value is compared to reference group. The socioeconomic index is a measure of the individual’s socioeconomic status and is derived from the specific occupation of the woman, producing a score between 10 and 90 [30]. Depression status was evaluated using the Edinburgh Postnatal Depression Scale. Customised birth weight centiles were calculated correcting for gestational age at birth, maternal ethnicity, maternal weight and height in early pregnancy, parity, and infant sex [37].

### Metabolic health characteristics

Box plots of measured values for each metabolic component in women with and without MetS are shown in S2 Fig. S2 Table shows the number and percentage of women with each metabolic abnormality, in women with and without MetS. In women with MetS, 88.3% of women had TGs above the IDF MetS cut-point, and 83.9% had raised glucose. All women, by definition, had a high WC. In comparison, in women who did not have MetS, 55.2% had high WC, 28.4% had raised glucose, and 20.8% had raised TGs.

S3 Fig shows the UpSet plot with the frequency for each combination of MetS components, and S4 Fig and S5 Fig show the UpSet plots for women with BMI <30 kg/m^2^ and ≥30 kg/m^2^, respectively. WC ≥80 cm was the most common single criterion, followed by the combination of WC and glucose. S3 Table shows the number and percentage of women with each pregnancy complication according to MetS.

### MetS and pregnancy outcomes

**Fig 1:**
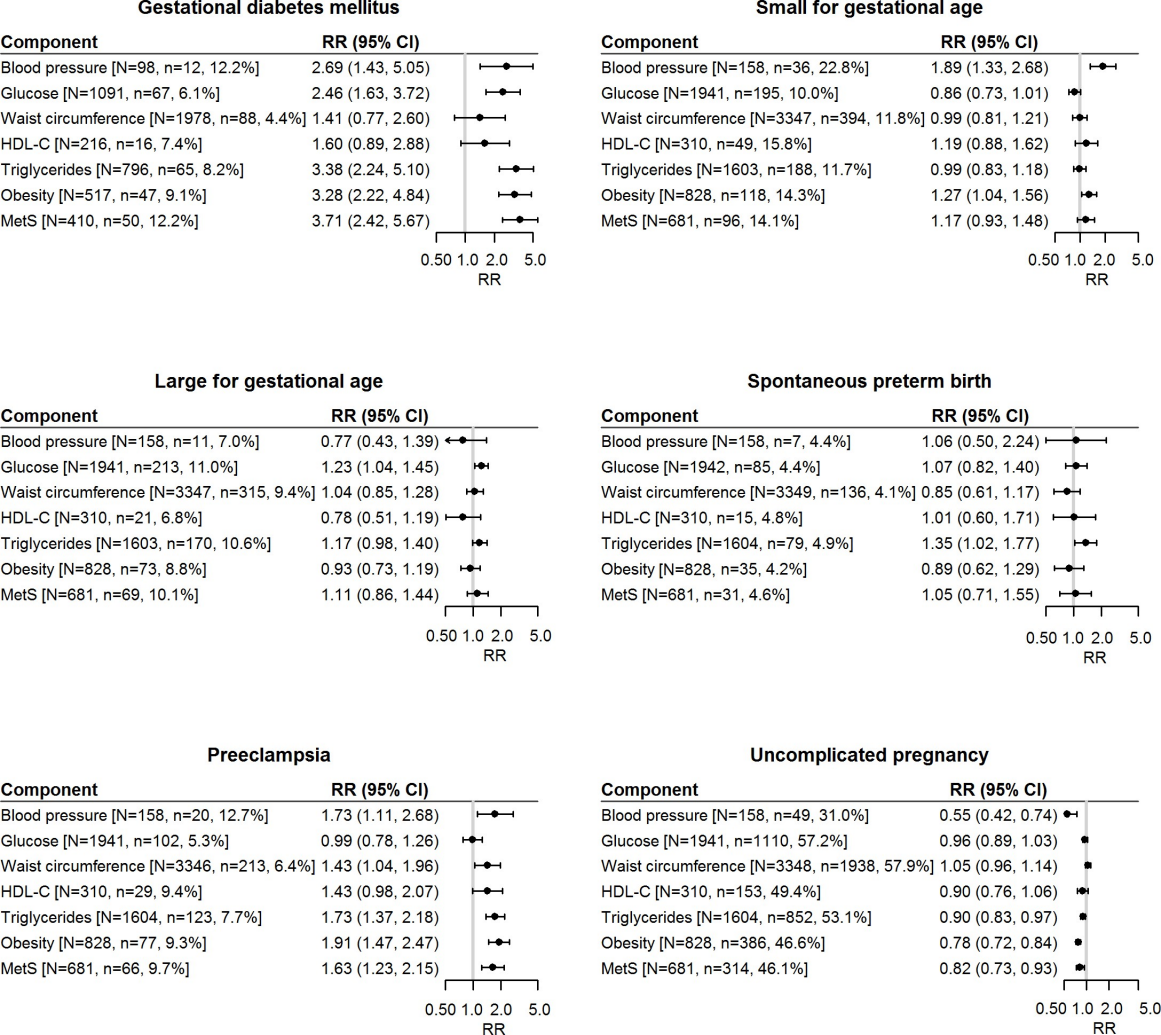
Forest plots for each pregnancy complication, the relative risk, the number of women with MetS or its individual components (*N*), and the number of women with the outcome (*n*). All models (except BMI on the forest plot, which represents BMI <30 kg/m^2^ as the reference compared to BMI ≥30 kg/m^2^) were adjusted for maternal BMI, age, study centre, ethnicity, socioeconomic index, physical activity, smoking status, depression status, and fetal sex. *N =* number of women with the metabolic abnormality; *n =* number of cases (i.e., women who had the outcome). HDL-C, high-density lipoprotein cholesterol; MetS, metabolic syndrome; RR, relative risk.

For each pregnancy complication, the relative risk, the number of women with MetS or its individual components (*N*), and the number of women with the outcome (*n*) are shown in Fig 1. Individual MetS components that increased risk for PE were raised BP (RR 1.73, 95% CI 1.11 to 2.68), raised TGs (RR 1.73, 95% CI 1.37 to 2.18), and high WC (1.43, 95% CI 1.04 to 1.96), after adjusting for maternal BMI, age, study centre, ethnicity, SEI, physical activity, smoking status, depression status, and fetal sex. Of the total cohort, the number of women with raised BP at 15 weeks’ gestation was small (*n =* 158); 20 (12.7%) of these women developed PE. In all, 3,346 women had a high WC, and 213 (6.4%) developed PE. Raised glucose and reduced HDL-C were not significantly associated with risk for PE. Women with MetS were at an increased risk for PE, with a relative risk of 1.63 (95% CI 1.23 to 2.15), and 66 (9.7%) of these women developed PE. In absolute terms, women with MetS had an adjusted excess risk of 2.52% (95% CI 1.51% to 4.11%), with a number needed to harm of 40.

Individual MetS components that increased risk for GDM were raised TGs (RR 3.38, 95% CI 2.24 to 5.10), raised BP (RR 2.69, 95% CI 1.43 to 5.05), and raised glucose (RR 2.46, 95% CI 1.63 to 3.72) (Fig 1). Ninety-eight women had raised BP, of whom 12 (12.2%) developed GDM; 796 women had raised TGs, of whom 65 (8.2%) developed GDM; and 1,091 women had raised glucose, of whom 67 (6.1%) developed GDM. High WC and reduced HDL-C were not associated with risk for GDM. Women with MetS were at the greatest increased risk for GDM, with a relative risk of 3.71 (95% CI 2.42 to 5.67). In absolute terms, women with MetS had an adjusted excess risk of 8.66% (95% CI 5.38% to 13.94%), with a number needed to harm of 12.

Risk for SGA was increased with raised BP (RR 1.89, 95% CI 1.33 to 2.68), but not with any of the other metabolic components, nor with MetS. For sPTB, risk was increased with raised TGs (RR 1.35, 95% CI 1.02 to 1.77), but not with any of the other metabolic components, nor with MetS. Raised glucose modestly increased risk for LGA (RR 1.23, 95% CI 1.04 to 1.45), but none of the other individual metabolic components, nor MetS, increased risk for LGA. MetS was associated with reduced probability of having an uncomplicated pregnancy (RR 0.82, 95% CI 0.73 to 0.93). In absolute terms, for having any pregnancy complication, women with MetS had an adjusted excess risk of 9.35% (95% CI 10.30% to 7.98%), with a number needed to harm of 11. Obesity (BMI ≥30 kg/m^2^) increased risk for GDM (RR 3.28, 95% CI 2.22 to 4.84), PE (RR 1.91, 95% CI 1.47 to 2.47), and SGA (RR 1.27, 95% CI 1.04 to 1.56).

**Fig 2:**
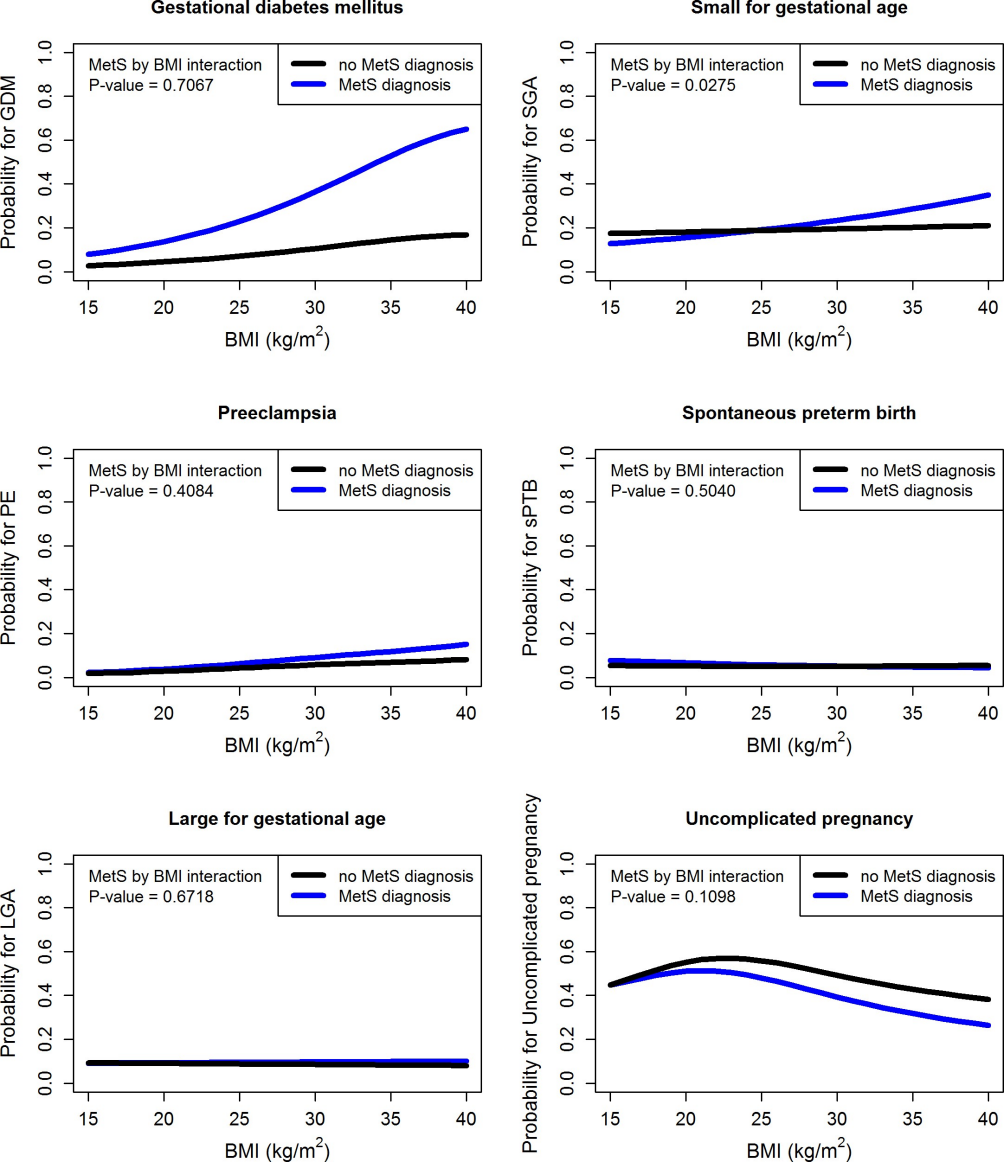
Predicted probability of each pregnancy outcome for women who did and did not have MetS and the interaction with BMI, estimated from the generalised additive model. Models adjusted for maternal BMI (as a spline), age, study centre, ethnicity, socioeconomic index, physical activity, smoking status, depression status, and fetal sex, with an interaction term between the metabolic groups and BMI. GDM, gestational diabetes mellitus; LGA, large for gestational age; MetS, metabolic syndrome; PE, preeclampsia; SGA, small for gestational age; sPTB, spontaneous preterm birth.

To determine the adjusted probability of pregnancy complications with increasing maternal BMI (non-linear) according to MetS, we used penalised spline (cubic spline with 4 knots) analysis in GAMs (Fig 2). As maternal BMI increases, the presence of MetS increases the estimated probability for GDM and SGA and also decreases the probability of an uncomplicated pregnancy.

## Discussion

### Main findings

To our knowledge, this is the first large, population-based, multi-centre prospective cohort study in low-risk nulliparous pregnant women to assess the association between MetS—rather than just its individual components—at 15 weeks’ gestation and pregnancy outcomes. We report that 12.3% (*n =* 684) of women had MetS, and these women were at an increased risk for PE and GDM, after adjustment for a range of demographic and lifestyle variables. Increasing BMI in combination with MetS also increased the estimated probability for GDM, and decreased the probability of an uncomplicated pregnancy. The findings build on the literature assessing the effects of individual metabolic health components or obesity on pregnancy complications, by using established clustering of abnormalities that are evident in non-obese pregnant women.

### Comparison to literature

There is an abundance of literature demonstrating that maternal obesity increases risk for pregnancy complications including GDM and PE [40–42] and also for babies born too small or large [43]. Some studies reporting on individual metabolic components, such as raised TGs or LDL-C or reduced HDL-C, and pregnancy complications show that these are associated with increased risk for GDM, PE, LGA, and sPTB [28]. In addition, raised maternal glucose, insulin resistance, and raised BP are associated with GDM [44,45], and pre-pregnancy WC is associated with GDM and LGA [46]. Examination of a composite of components (MetS) and its association with pregnancy complications extends these earlier studies assessing individual components.

Currently, all obese pregnant women are considered to be at the same increased risk for GDM. However, only 15%–30% of these women will develop the disorder [47]. Recently, in a sample of 1,303 obese pregnant women, a model based on clinical and anthropometric variables (e.g., age and systolic BP) could modestly predict GDM (AUC 0.71), with the AUC increased to 0.77 with the addition of candidate biomarkers such as random glucose and TGs [48]. However, while obese women are at higher risk for GDM than their lean or overweight counterparts, 7%–15% of lean women may still develop GDM [49,50]. Our results suggest that assessment of MetS in all pregnant women may provide information on risk for GDM that extends beyond BMI. Although MetS conferred a slightly greater risk for GDM than glucose, TGs, and WC assessed individually, the impact of MetS as a function of a combination of components or exclusion of certain components requires investigation in other pregnancy cohorts. Additionally, whether MetS improves prediction of risk of GDM above that of raised glucose alone needs to be examined, and should be assessed in women who are obese or not. This will help determine any applicability of diagnosing MetS in the antenatal setting, in addition to standard BMI and glucose measurements.

Recent figures estimate that PE affects 2%–7% of pregnancies and occurs more frequently in nulliparous women [51]. Women who have experienced PE are at a more than 2-fold increased risk for future CVDs such as hypertension and ischemic heart disease [52]. Early identification of women at risk for PE remains one of the major focuses of antenatal care. We found that MetS, a risk factor for future CVD, increased the risk for PE, but in combination with increasing BMI did not demonstrate greater risk. We found that a smaller number of women had raised BP (*n =* 198) than had MetS (*n =* 681), but a similar proportion of each went on to develop PE (~10%). Future work comparing whether MetS provides risk information beyond that of raised BP would be useful.

### Health implications and clinical relevance

Traditionally, antenatal care pathways are based on individual needs, history, and specific risk factors such as obesity. Despite the best care, a number of women still go on to develop pregnancy complications. Not all available screening tests are offered to all women, and some are only offered to those who are deemed at risk. Testing for impaired glucose tolerance in early pregnancy is rarely provided but may be undertaken before 24 weeks’ gestation if the woman has risk factors. Some guidelines recommend that women have their risk for GDM evaluated by assessment of maternal risk factors followed by an oral glucose tolerance test when required. Furthermore, in nulliparous women, risk assessment for other pregnancy complications such as PE is difficult as there is no maternal history and no widely adopted screening test.

Our findings suggest that MetS diagnosed in early pregnancy may be used to broadly identify women at increased risk for pregnancy complications. The utility of MetS in predicting pregnancy complications, as a complementary component to standard routine antenatal care and compared to standard risk factors, requires exploration. Future studies are also encouraged to define clear metabolic health phenotypes that will produce the optimal probability threshold for each outcome.

### Strengths and limitations

The major strength of this study is that it used a population-based prospective cohort design that included a large number of nulliparous women across 6 centres in 4 countries. Participants comprised a clearly defined population of nulliparous low-risk women with no pre-existing disease, which should be considered in study design for other populations. Risk estimates may potentially be underestimated for the general population as the participants in this study are at low risk for pregnancy complications compared to the general population of pregnant women. The study captured 5,530 pregnant women for whom complete information on plasma and other maternal metabolic variables and pregnancy and neonatal outcomes was available. These were ascertained using rigorous assessment methods standardised across recruiting centres, resulting in negligible information bias. A key strength is the assessment of individual and composite MetS compoments and pregnancy complications, which have not been thoroughly investigated to date.

There are limitations in this study. Currently, there are several different definitions for MetS in the adult population as different numbers and types of metabolic variables are used, as well as different cut-off values, between studies [10,11]. However, there are no definitions at all specifically for pregnancy, and, despite pregnancy being a hyperlipidemic state, there is no agreed threshold for pathological hyperlipidemia in pregnancy. We used the IDF definition for MetS for adults, as previous studies have demonstrated no remarkable increase in lipids in first trimester of pregnancy [53], and it is unlikely that WC would have significantly increased at the time of recruitment at 15 weeks’ gestation. There may be inherent limitations with using non-fasting compared to fasting metabolic components, particularly for TGs and glucose; however, an increased risk for PE and GDM has been demonstrated using fasting [25,54,55] and non-fasting [56–61] lipids. Universal screening for GDM in the UK and Ireland was not available at the time of recruitment, and screening was only undertaken for women who were deemed at risk; thus, we only included data for the centres with universal screening (*n =* 3126) for this outcome. A recent study has highlighted the importance of early screening for GDM in obese women, demonstrating differences in metabolic profile between obese women who do and do not develop GDM [45]. Finally, assessment of MetS was made in the first trimester, and it is unclear whether metabolic components may have altered since conception.

### Generalisability

Around 90% of the study participants were white; thus our results are likely generalisable to populations of pregnant women who are white and considered to be at low risk for disease. The results may have limited applicability to other ethnic groups with potentially different patterns of metabolic health and known differences in risk for pregnancy complications. It is also unclear whether these results are generalisable to women of all parities. However, women who have had a complication in a previous pregnancy may modify their behaviours, and clinicians would monitor them more closely in subsequent pregnancies.

## Conclusion

We did not compare the impact of individual metabolic components with that of MetS as a composite, and therefore cannot conclude that MetS is better at identifying women at risk. However, more than half of the women who had MetS in early pregnancy developed a pregnancy complication compared with just over a third of women who did not have MetS. Furthermore, while increasing BMI increases the probability of GDM, the addition of MetS exacerbates this probability. Further studies are required to determine if individual MetS components act synergistically or independently. It is currently unclear whether women in this study had an adverse metabolic profile prior to pregnancy, and, therefore, studies to assess and monitor changes in metabolic profile pre-conception and throughout pregnancy may be helpful. Young women identified as having poor metabolic health in pregnancy are at increased risk for pregnancy complications, and these women will likely need metabolic follow-up in the years after their pregnancy.

## Supporting information

S1 STROBE Checklist STROBE checklist. (DOCX)

S1 Fig Study flow chart. *Groups not mutually exclusive (total *n =* 98 with missing measurements).
(TIF)

S2 Fig Box plots demonstrating median (interquartile range) for each MetS component, according to MetS status. Dotted line represents cut-point for IDF definition for MetS components: reduced HDL-C (<1.29 mmol/l [<50 mg/dl]), raised TGs (≥1.70 mmol/l [≥150 mg/dl]), raised plasma glucose (≥5.6 mmol/l), raised systolic BP (≥130 mm Hg) or raised diastolic BP (≥85 mm Hg), and high WC (≥80 cm).
(TIF)

S3 Fig UpSet plot displaying the frequency for each combination of MetS components. (TIFF)

S4 Fig UpSet plot displaying the frequency for each combination of MetS components in women with BMI <30 kg/m^2^. (TIFF)

S5 Fig UpSet plot displaying the frequency for each combination of MetS components in women with BMI ≥30 kg/m^2^. (TIFF)

S1 Table Missing data summary. (XLSX)

S2 Table Number and percentage of women with metabolic abnormalities, according to MetS status. (XLSX)

S3 Table Crude numbers and percentages of women with each pregnancy complication, in women with and without MetS. (XLSX)

S1 Text Study protocol and analysis plan. (DOC)

## Abbreviations

AUC: area under the curve
BP: blood pressure
CVD: cardiovascular disease
GAM: generalised additive model
GDM: gestational diabetes mellitus
HDL-C: high-density lipoprotein cholesterol
IDF: International Diabetes Federation
LDL-C: low-density lipoprotein cholesterol
LGA: large for gestational age
MetS: metabolic syndrome
PE: preeclampsia
RR: relative risk
SCOPE: Screening for Pregnancy Endpoints
SEI: socioeconomic index
SGA: small for gestational age
sPTB: spontaneous preterm birth
T2DM: type 2 diabetes mellitus
TGs: trigycerides
WC: waist circumference
