# Supplementary figures and images for: Metabolic syndrome in pregnancy and risk for adverse pregnancy outcomes: A prospective cohort of nulliparous women
Source: PLoS Med. 2018 Dec 4;15(12):e1002710. doi: 10.1371/journal.pmed.1002710 (PMC6279018; doi:10.1371/journal.pmed.1002710)

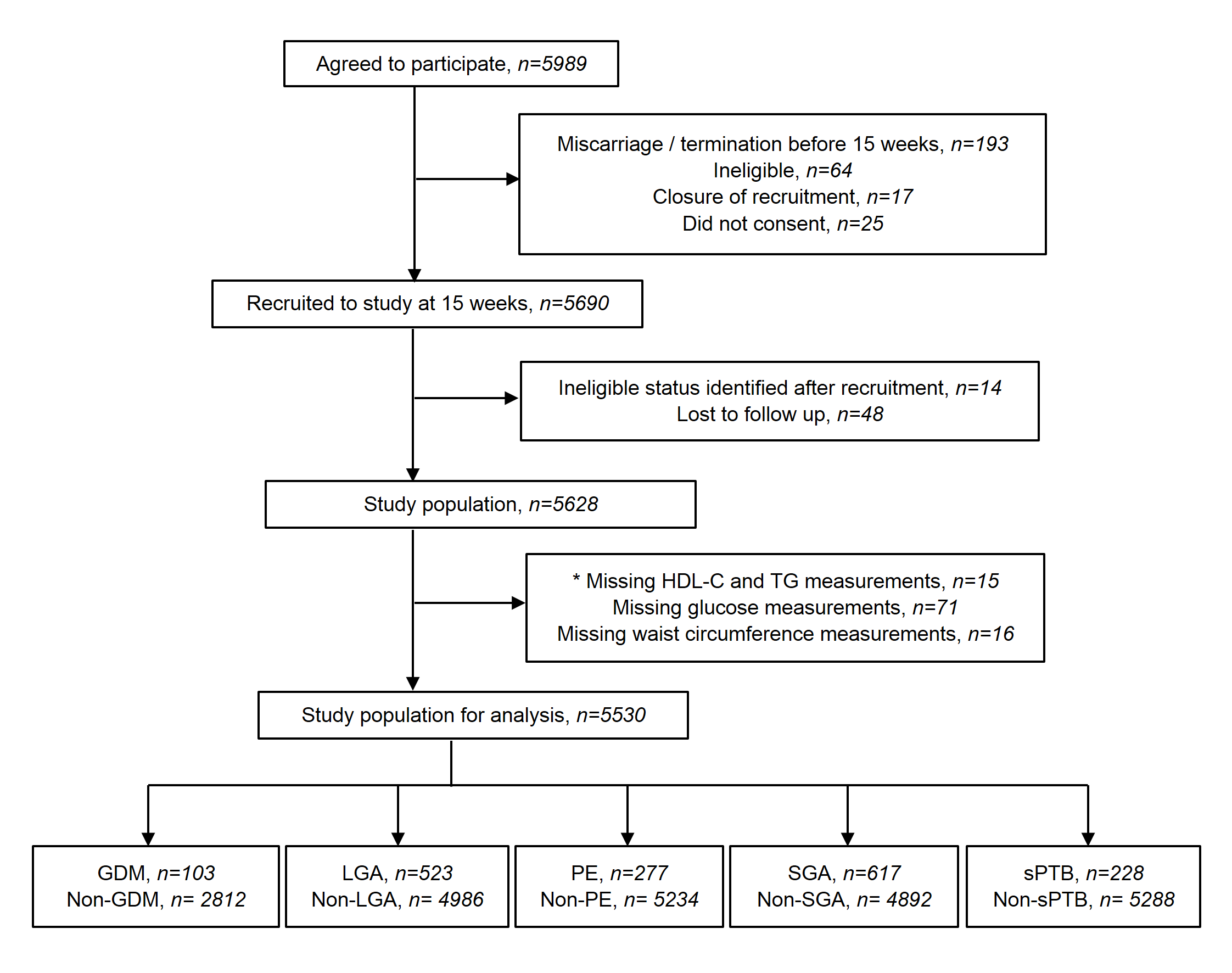

Supplement: S1 Fig — *Groups not mutually exclusive (total n = 98 with missing measurements). (TIF) [file pmed.1002710.s002.tif]

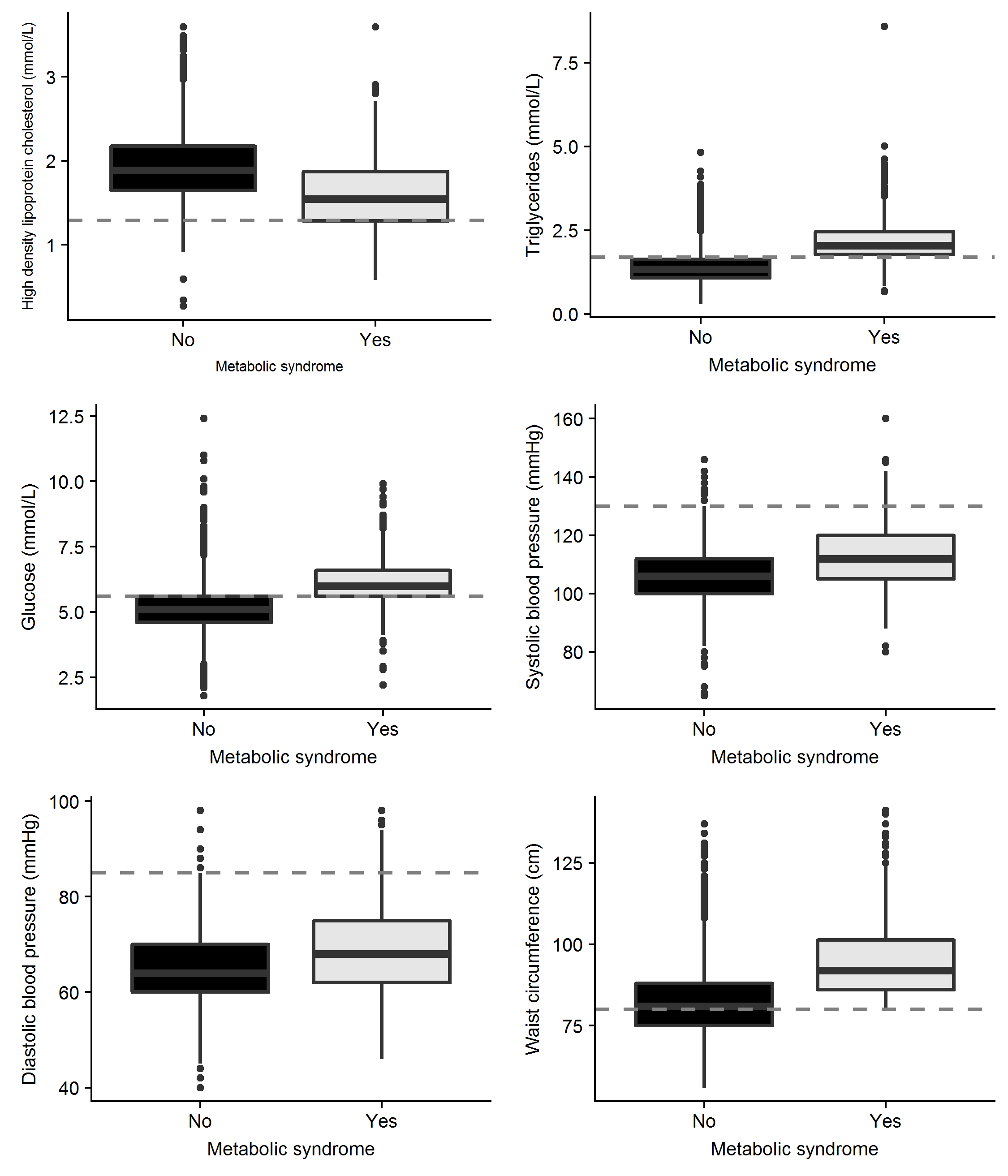

Supplement: S2 Fig — Dotted line represents cut-point for IDF definition for MetS components: reduced HDL-C (<1.29 mmol/l [<50 mg/dl]), raised TGs (≥1.70 mmol/l [≥150 mg/dl]), raised plasma glucose (≥5.6 mmol/l), raised systolic BP (≥130 mm Hg) or raised diastolic BP (≥85 mm Hg), and high WC (≥80 cm). (TIF) [file pmed.1002710.s003.tif]

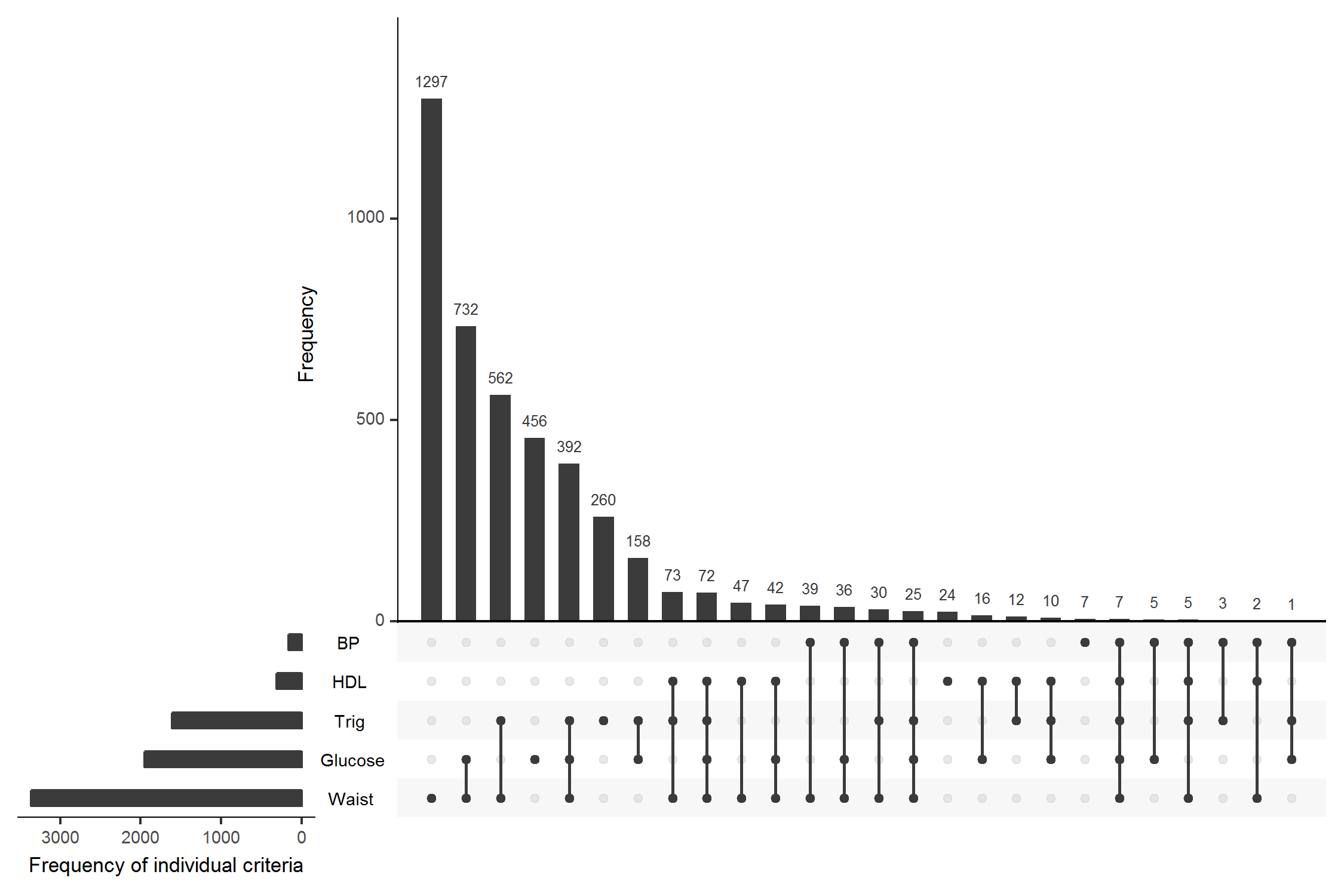

Supplement: S3 Fig — (TIFF) [file pmed.1002710.s004.tiff]

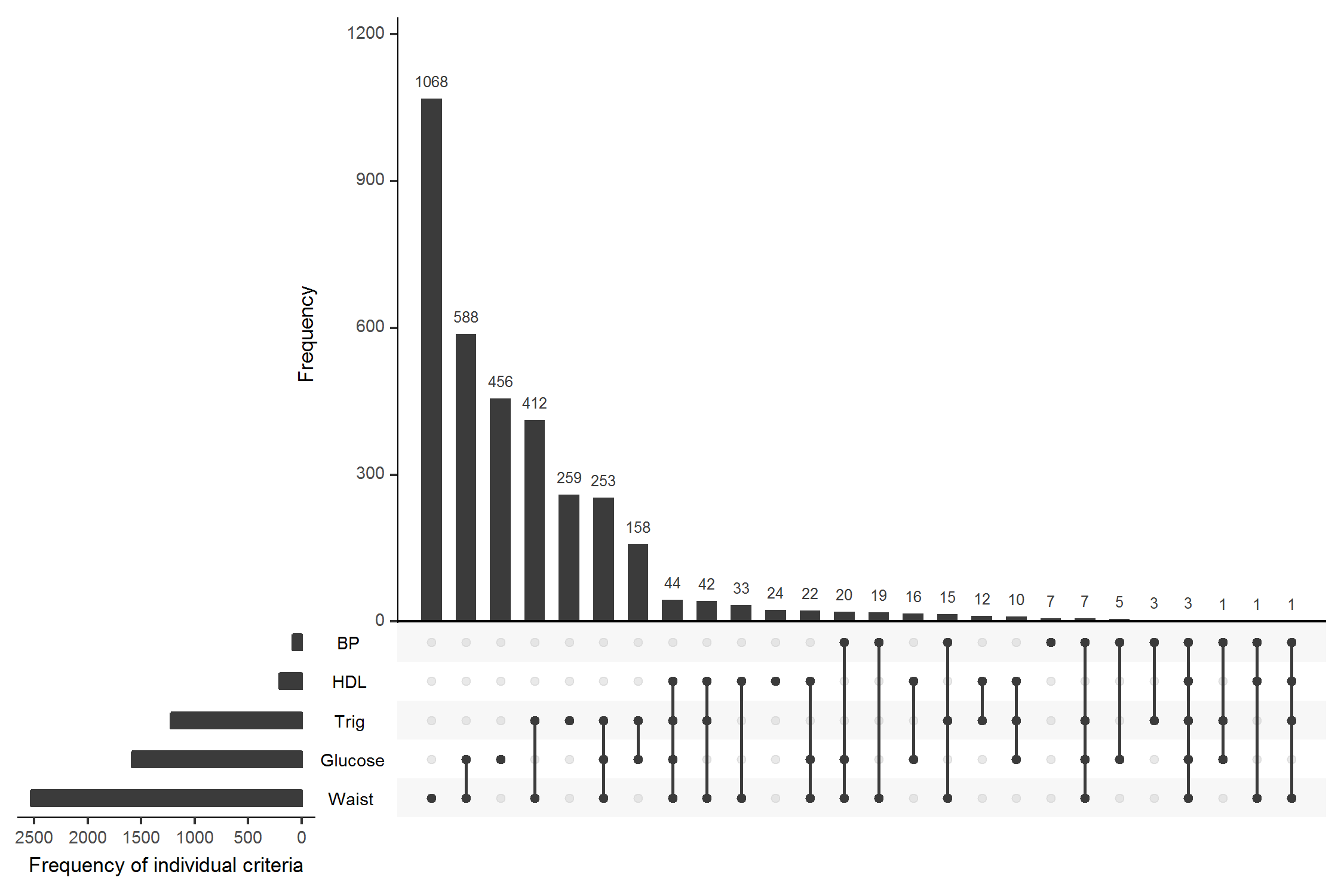

Supplement: S4 Fig — (TIFF) [file pmed.1002710.s005.tiff]

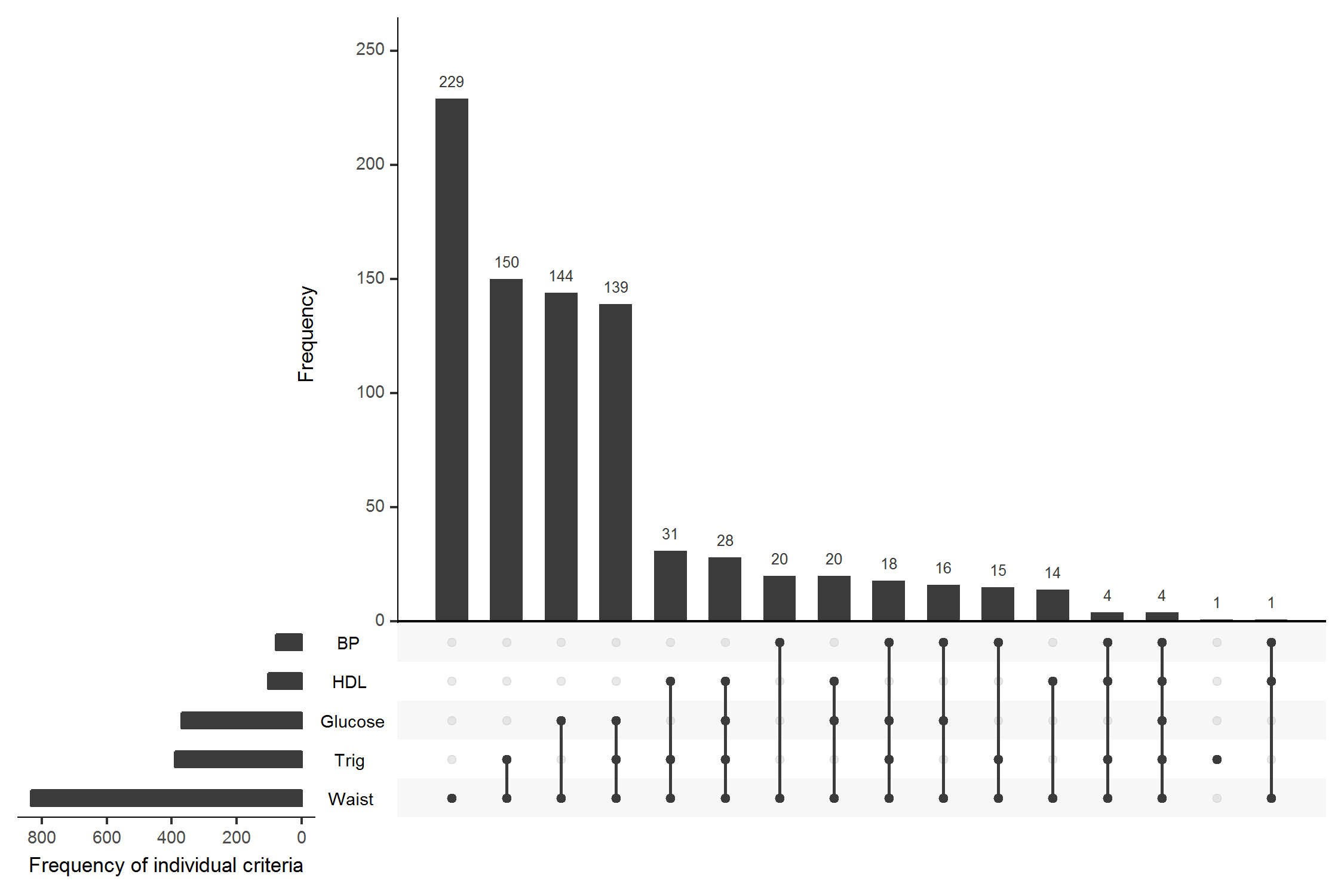

Supplement: S5 Fig — (TIFF) [file pmed.1002710.s006.tiff]
