# Supplementary material for: Metabolic syndrome in pregnancy and risk for adverse pregnancy outcomes: A prospective cohort of nulliparous women
Source: PLoS Med. 2018 Dec 4;15(12):e1002710. doi: 10.1371/journal.pmed.1002710 (PMC6279018; doi:10.1371/journal.pmed.1002710)
Supplement: S1 Text — (DOC) [file pmed.1002710.s010.doc]

**APPLICATION FORM**

**SCOPE RESEARCH PROJECT**

**RAF Number: 3.80**

**Project Title: Metabolic phenotypes and pregnancy outcomes.**

**Researcher(s): Dr Jessica Grieger, Prof Claire Roberts, Dr Tina Bianco-Miotto, Prof Gus Dekker, Prof Lesley McCowan**

**Date of Submission: 17/09/2015**

Mark X in each centre involved in the research

|  | SCOPE Centres involved in research | SCOPE Biobanks Data involved in research | SCOPE Biobanks Specimen use in research |
| --- | --- | --- | --- |
| Auckland | X | X |  |
| Adelaide | X | X |  |
| Manchester | X | X |  |
| Kings | X | X |  |
| Leeds | X | X |  |
| Cork | X | X |  |

**Funding required to do project yes no** (delete as appropriate)

| **Funding Status** | **Yes** | **No** | **N.A.** | **Funder/Amount funded/Date** |
| --- | --- | --- | --- | --- |
| Applied for funding |  |  |  | Can cover this on existing funds for statistician and Jess’s ECR Fellowship |
| Planning to apply for funding |  |  |  |  |
| Funder/Potential Funder |  |  |  |  |
| Date expect to hear outcome of funding application |  |  |  |  |
| Date heard outcome of application |  |  |  |  |
| Funded |  |  |  |  |
| Amount funded |  |  |  |  |

**Ethics**

Ethical Approval is required for all SCOPE clinical centres, and women consent prior to participating. Most projects should be covered by this consent, but there maybe occasion when additional ethical approval is required.

**Ethics Approval complete**  **yes**

**Collaboration with Commercial Institution or Academic Inst. outside SCOPE Consortium outside**

**YES**

**If yes, please complete this table:**

| **Commercial Details** | **Complete details** |
| --- | --- |
| Name of Company/ Academic Institution | None as yet |
| Type of Collaboration |  |
| IP ownership solved |  |
| If IP ownership solved, please summarise solution |  |
| If IP ownership not solved, summarise issues |  |

Note: If more than 1 institution involved please copy the rows in table and complete for each institution

**Research Proposal**

**The impact of metabolic phenotype on maternal and perinatal outcomes.**

**Summary Abstract**

Using the SCOPE databases, we will determine whether maternal metabolic phenotype is associated with maternal and neonatal outcomes including preeclampsia, gestational diabetes, spontaneous preterm delivery, small- and large for gestational age, intrauterine growth restriction, and macrosomia (birth weight ≥4000 g).

**Background:**

The global increase in overweight and obesity (National Institute of Diabetes and Digestive and Kidney Diseases. 2012; Australian Bureau of Statistics. 2013; World Health Organisation. 2015) reflects the growing prevalence of women entering pregnancy who are also overweight/obese (Callaway, Prins et al. 2006; Athukorala, Rumbold et al. 2010). Maternal BMI is a proxy measure of nutritional status (Dodd, Grivell et al. 2011; Kongubol and Phupong 2011; Ovesen, Rasmussen et al. 2011; Gaudet, Tu et al. 2012) and numerous epidemiological studies have reported that maternal overweight (i.e., BMI ≥25 kg/m^2^) increases the risk for large for gestational age and fetal macrosomia (reviewed in (Grieger and Clifton 2015)). Maternal obesity also can be associated with undernutrition in the mother as well as the fetus (reviewed in (Correia-Branco, Keating et al. 2014)), with consequences to the neonate including intrauterine growth restriction and altered epigenetic programming, which has been linked to later life disease in the offspring (Hochberg, Feil et al. 2011).

One of the underlying features regarding body weight, specifically, obesity, is metabolic health. Obesity in itself is associated with increased risk of developing co-morbidities including metabolic syndrome, type 2 diabetes mellitus and cardiovascular disease (Chang and Neu 2015). However, the obese phenotype may exist in the absence of metabolic abnormalities such as dyslipidaemia, insulin resistance, hypertension and an unfavourable inflammatory profile (Karelis and Rabasa-Lhoret 2008; Stefan, Kantartzis et al. 2008; Wildman, Muntner et al. 2008), and such individuals have been termed “metabolically healthy obese”. Yet, not all non-obese individuals present a healthy metabolic profile, implying there is a range of metabolic health phenotypes which would likely impact a range of metabolic and cardiovascular outcomes. Unfortunately to date, there is no agreed definition regarding metabolic syndrome (Phillips 2013), such that there is a large discrepancy with adverse metabolic phenotype, with prevalence rates in adults varying between 3.3-32.1% in men and between 11.4-43.3% in women, depending on the criteria used (Velho, Paccaud et al. 2010). Moreover, the inclusion of BMI/obesity in these definitions has not been assessed.

Some studies have investigated the association between individual lipid measurements on maternal and neonatal outcomes (Barrett, Dekker Nitert et al. 2014; Hooijschuur, Ghossein-Doha et al. 2015), however the impact of maternal metabolic health as a clustering of adverse metabolic parameters on these outcomes has not been currently assessed. This would be important given that maternal nutrition impacts fetal growth (Pereira, Moyce et al. 2014; Grieger and Clifton 2015); and dietary manipulation is associated with changes in blood lipids, glucose metabolism, blood pressure, and anthropometric measurements (Wood, Kabagambe et al. 2011; Hu, Mills et al. 2012; Schwingshackl and Hoffmann 2013; Schwingshackl and Hoffmann 2013; Schwingshackl and Hoffmann 2013), which all encompass metabolic health and impact later chronic disease.

**Aims**

1) To determine the metabolic phenotype of pregnant women participating in SCOPE (Screening for Pregnancy Endpoints Study).

2) To determine whether metabolic phenotype is associated with maternal and neonatal outcomes including preeclampsia, gestational diabetes, spontaneous preterm delivery, small- and large for gestational age, intrauterine growth restriction, and macrosomia (birth weight ≥4000 g).

**Hypotheses**

1) Women who are metabolically unhealthy and obese (i.e. worst criteria) will be at greater risk toward developing adverse maternal or neonatal outcomes compared to women who are metabolically healthy and not obese (i.e. best criteria).

2) Women who are metabolically unhealthy and not obese will be at greater risk toward developing adverse maternal or neonatal outcomes compared to women who are metabolically healthy and obese.

**Significance**

Abnormal maternal metabolic markers adversely affect maternal and neonatal outcomes including increased risk for gestational diabetes, preeclampsia, large for gestational age and preterm delivery. Results from this study will be the first to demonstrate that identification of metabolic phenotypes, defined by a clustering of metabolic abnormalities during the first half of pregnancy is necessary so that targeted interventions to improve metabolic health will subsequently optimise maternal and perinatal outcomes.

**Study Design:**

Cohort study of all women recruited to SCOPE (n=5628).

**Methods**

*Population and data collection*

Participants recruited to the Screening for Pregnancy Endpoints Study (SCOPE) were healthy, nulliparous women recruited from Auckland (New Zealand), Adelaide (Australia), Cork (Ireland), Leeds, London and Manchester (UK) (n=5628). Data collection was obtained by a research midwife at 14-16 weeks gestation and consisted of demographic information, smoking, family, medical and gynaecological history, as well as blood pressure, height and weight (to determine BMI), waist circumference, and a non-fasting blood sample. Blood pressure was used to calculate mean arterial pressure (MAP, 2/3 diastolic pressure plus 1/3 systolic blood pressure) and used in the metabolic health criteria (see below). Diet and supplementation use was ascertained at the same time point, as well as at 20 weeks gestation. Pre-pregnancy and first trimester data were ascertained at the 15 weeks’ visit. For the purpose of this secondary analysis, non-fasting serum blood measurements taken at 14-16 weeks gestation included total cholesterol, high density lipoprotein (HDL)-cholesterol, low density lipoprotein (LDL)-cholesterol, triglycerides, and a random glucose test.

*Definition of metabolic health*

There is currently no one single definition for metabolic health in the adult population, however, at least 3 abnormalities have typically been used where 6 or 7 criteria are available (Phillips 2013). Therefore, based on the above, lipids (i.e. total cholesterol, HDL-C, LDL-C, and triglycerides), waist circumference, random glucose, and mean arterial pressure were included in our definition of metabolic health with ≥4 out of 8 criteria defining metabolically unhealthy. To categorise women as metabolically healthy or unhealthy, normal/abnormal lipid levels will be used. Thus, the following measures were dichotomised into ‘normal’ and ‘abnormal’ categories, where abnormal was defined as: i) total cholesterol ≥5 mmol/L ii) LDL cholesterol ≥2.5 mmol/L; iii) HDL cholesterol <1.4 mmol/L; iv) triglycerides ≥1.69 mmol/L; v) random glucose (≥8.5 mmol/L), vi) mean arterial pressure (≥93.3), and vii) waist circumference (>80cm). Based on the above “abnormal” categories, 4 categories relevant to metabolic health were created. That is, metabolically healthy, not obese (best outcome: 0-2 abnormal criteria and a BMI <30 kg/m^2^); metabolically unhealthy, not obese (3-7 abnormal criteria and a BMI <30 kg/^2^); metabolically healthy and obese (0-2 abnormal criteria and a BMI ≥30 kg/m^2^); and metabolically unhealthy, and obese (poorest outcome: participants had 3-7 abnormal criteria and a BMI ≥30 kg/m^2^).

1. We will use the SCOPE database to determine the percentage of women according to metabolic phenotype.

2. To determine whether maternal metabolic phenotype is associated with maternal and neonatal outcomes including preeclampsia, gestational diabetes, spontaneous preterm delivery, small- and large for gestational age, intrauterine growth restriction, and macrosomia (birth weight ≥4000 g).

**Primary outcomes:**

- Gestational diabetes
- Pregnancy-induced hypertension
- Preeclampsia
- Spontaneous Pre-term birth
- Small for gestational age infants
- Large for gestational age infants
- Intrauterine growth restriction

**Secondary outcomes:**

- Birth weight
- Cesarean delivery
- Operative vaginal delivery
- Miscarriage
- Length of gestation

**Demographic, anthropometric, lifestyle and non-lifestyle related factors**

- Age
- Ethnicity
- Country
- Socioeconomic status
- Education
- Occupation
- Income
- Spontaneous or assisted reproductive technology pregnancy
- Blood pressure
- Infertility status
- Family history of pregnancy complications, type 2 diabetes and cardiovascular disease
- Weight and BMI
- Waist circumference and waist to hip ratio
- Skinfolds
- Gestational weight gain
- Medication use during pregnancy
- Infertility treatment
- Smoking
- Alcohol intake
- Micronutrient intake
- Diet (vegetarian status, oily fish consumption, fruit consumption, green leafy vegetable consumption, non-core food consumption)
- Physical activity
- Psychological outcomes (self-reported depression, perceived stress scale, STAI, depression scale)
- Lifestyle questionnaire

Type(s) and volume of specimens required:

| Specimen Type (e.g., EDTA plasma) | Gestation of Specimen (15w, 20w or Time of disease/late control) | Number of Aliquots per woman* | Total of Aliquots in Expt |
| --- | --- | --- | --- |
| NIL |  |  |  |
|  |  |  |  |
|  |  |  |  |

*All blood specimens 250ul aliquots, urine 0.9ml aliquot

**Modifications based on the comments from editors and reviewers:**

Following the suggestion of reviewers and editors, we have re-analysed the data using the established International Diabetes Federation criteria for metabolic syndrome rather than data driven values (blood pressure and glucose). In light of editors comments, we did not stratify for obesity but included BMI as a continuous variable as a confounder in our analyses.

**References**

**Other Comments**:

We have sufficient funds to complete the statistical analyses.

**Planned commencement date:**

September 2015

**Expected finish date:**

April 2016

**Consortium appendix required no**

**Consortium appendix completed yet to be completed**

**SCOPE office to complete**

SCOPE Project Number 3.80_______________

**Lead Reviewer:**

**Study: Approved Not Approved**

| **Approval Process** | **No or N.A.** | **Yes** | **Date** |
| --- | --- | --- | --- |
| Received completed application |  |  | **17/09/2015** |
| Emailed to Research Group for review |  |  |  |
| Approved by Research Group |  |  |  |
| Summary abstract emailed to Scientific Committee |  |  |  |
| Approved by Scientific Committee |  |  |  |
| RAF resubmitted to Research Group for re assessment |  |  |  |
| Resubmitted RAF –  approved by Research Group |  |  |  |
| Summary abstract emailed to Scientific Committee |  |  |  |
| Approved by Scientific Committee |  |  |  |
| Summary emailed to SCOPE Board |  |  |  |
| Requires formal assessment by SCOPE Board |  |  |  |
| Approved by SCOPE Board |  |  |  |
| Applicant notified of project approval |  |  |  |
| Consortium appendix requested to be completed |  |  |  |
| Appendix returned to SCOPE office |  |  |  |
| Appendix signed by required parties |  |  |  |

| **Project Progress** | **Date/Details** |
| --- | --- |
| Commenced study |  |
| Completed study |  |
| Published | yes / no |
| Date published |  |
| Journal details |  |
| Filed patent | yes / no |
| Date filed patent |  |
| Details of patent(s) e.g. filed where, patent number |  |
| Date Patent Issued |  |
